# Down-Regulation of CTLA-4 by HIV-1 Nef Protein

**DOI:** 10.1371/journal.pone.0054295

**Published:** 2013-01-23

**Authors:** Mohamed El-Far, Catherine Isabelle, Nicolas Chomont, Martin Bourbonnière, Simone Fonseca, Petronela Ancuta, Yoav Peretz, Younes Chouikh, Rabih Halwani, Olivier Schwartz, Joaquín Madrenas, Gordon J. Freeman, Jean-Pierre Routy, Elias K. Haddad, Rafick-Pierre Sékaly

**Affiliations:** 1 Centre de Recherche du Centre Hospitalier de l'Université de Montréal (CRCHUM), Hôpital St-Luc, Montréal, Québec, Canada; 2 Laboratoire d'Immunologie, Département de Microbiologie et d'Immunologie, Université de Montréal, Montréal, Québec, Canada; 3 Vaccine and Gene Therapy Institute Florida, Port St. Lucie, Florida, United States of America; 4 Prince Naif Center for Immunology Research and Department of Pediatrics, College of Medicine, King Saud University, Riyadh, Saudi Arabia; 5 Virus and Immunity Group, Department of Virology, Institut Pasteur, Paris, France; 6 Department of Microbiology and Immunology, McGill University, Montreal, Canada; 7 Dana Farber Cancer Institute, Harvard Medical School, Boston, Massachusetts, United States of America; 8 Chronic Viral Illness Service and Division of Hematology, Royal Victoria Hospital, McGill University Health Centre, McGill University, Montréal, Canada; University of Houston, United States of America

## Abstract

HIV-1 Nef protein down-regulates several cell surface receptors through its interference with the cell sorting and trafficking machinery. Here we demonstrate for the first time the ability of Nef to down-regulate cell surface expression of the negative immune modulator CTLA-4. Down-regulation of CTLA-4 required the Nef motifs DD175, EE155 and LL165, all known to be involved in vesicle trafficking. Disruption of the lysosomal functions by pH-neutralizing agents prevented CTLA-4 down-regulation by Nef, demonstrating the implication of the endosomal/lysosomal compartments in this process. Confocal microscopy experiments visualized the co-localization between Nef and CTLA-4 in the early and recycling endosomes but not at the cell surface. Overall, our results provide a novel mechanism by which HIV-1 Nef interferes with the surface expression of the negative regulator of T cell activation CTLA-4. Down-regulation of CTLA-4 may contribute to the mechanisms by which HIV-1 sustains T cell activation, a critical step in viral replication and dissemination.

## Introduction

HIV-1 regulatory viral proteins such as Nef, Vif, Vpr and Vpu create a cellular environment that is favorable for viral replication and dissemination [1]. Of particular importance, Nef plays a critical role in modulating the cellular microenvironment required for efficient viral replication by down-regulating multiple cell surface molecules through its interference with the intracellular sorting machinery [2], [3]. Nef-mediated down-regulation of CD4, the major HIV receptor, prevents superinfection during the early and late stages of infection as well as formation of the viral Env protein/CD4 oligomers during the budding process [4], [5], [6], [7], [8], [9]. Nef also down-regulates MHC class I molecules in infected cells, likely preventing their killing by cytotoxic CD8 T cells [10]. Expression of Nef enhances HIV-1 production by interacting with PI3k and p21-activated kinase2 (PAK2) [11], [12]. In addition, Nef is known to modulate several pathways of cell signaling and protects infected cells from apoptosis through the phosphorylation and inactivation of Bad, a proapoptotic member of the Bcl-2 protein family [13]. Moreover, the presence of Nef alters T cell activation through its interaction with the T cell-specific tyrosine kinase Lck *via* a conserved proline-rich repeat sequence {(PxxP)4} [14], [15], [16].

Nef has also been reported to play a critical role in the early activation of infected cells by sensitizing TCR to stimulation, thereby promoting secretion of the major T cell growth factor IL-2 and HIV replication [17], [18]. However, stimulation of T cells *via* TCR and CD28 leads to the up-regulation of molecules such as CTLA-4, which are known to negatively regulate cell activation [19] and potentially HIV replication. CTLA-4 is a cell surface protein that interacts with its ligands CD80 (B7-1) and CD86 (B7-2) expressed on APCs and stops T cell activation and IL-2 production [20], [21]. CTLA-4 is also essential for the suppressive functions of Tregs [22] and the induction of indoleamine 2,3-dioxygenase (IDO) in tolergenic dendritic cells [23]. CTLA-4 is found mainly as an intracellular protein that resides in endocytic vesicles and secretory granules [24], [25]. Surface expression of CTLA-4 is regulated by tyrosine motifs embedded within its cytoplasmic tail and mediate CTLA-4 binding to the μ2 subunit of the adaptor sorting protein AP2. Following TCR stimulation, these tyrosine motifs become phosphorylated and prevent AP2-mediated CTLA-4 internalization leading to CTLA-4 accumulation on the cell surface [26], [27], [28], [29].

The mechanism(s) underlying sustained HIV-1 replication in activated T cells that express high levels of molecules such as CTLA-4 have yet to be elucidated. Here, we show that HIV-1 Nef protein down-regulates surface and total expression of CTLA-4 by targeting this negative molecule to lysosomal degradation.

## Materials and Methods

### Cells

The 293T and HeLa cell lines were obtained from ATCC. Cells were kept in DMEM medium, 10% FCS and penicillin/streptomycin (Gibco-Life Technologies) and maintained at 37°C and 5% CO_2_.

### Antibodies

For FACS analysis on 293T cells we used anti-CD4 PE antibody (BD) and biotinylated Goat anti-CTLA-4 antibody from R&D Systems (used in combination with Streptavidine-APC). Anti-Nef and anti-CTLA-4 antibodies used in Western blot analysis were homemade by injecting rabbits with full length of these proteins fused to GST. Both homemade anti-CTLA-4 and anti-Nef polyclonal antibodies recognized the purified forms of GST-fused CTLA-4 and Nef proteins that were used to immunize rabbits. These antibodies also reacted positively with CTLA-4 and Nef transfected but not un-transfected cells and recognized proteins with the expected molecular weights of 30–34 kD for CTLA-4 and 27 kD for Nef. Anti-human CD4 antibody for Western blotting was purchased from RDI and mouse anti-*β*-actin antibody was purchased from Sigma. For confocal microscopy experiments, all secondary rabbit anti-mouse antibodies, anti-FLAG conjugated with Alexa 568, mouse anti-goat conjugated with Alexa 488, and transferrin labeled with Alexa 633 were purchased from Molecular probes.

### Nef and CTLA-4 Vectors

The CTLA-4 eukaryotic vector was synthesized by inserting the CTLA-4 coding sequence by PCR into the EcoR1 and Xba1 restriction sites of pCDNA3 (Invitrogen). The primers used were CTLA-4 5p/EcoRI: 5′- CATCGAATTCATGGCTTGCCTTGG-3′ and CTLA-4 3p/XbaI: 5′-TGCTCTAGATCAATTGATGGG-3′. The CD4 eukaryotic expression vector was generated in our laboratory. The CD4 cDNA was cloned in SR-alpha expression vector under the control of the CMV promoter. The Nef-FLAG expression vector used was pCMVtag4A vector obtained from Invitrogen. Nef was cloned using 5′EcoR1 and 3′HindIII restriction sites. The primers used were: NefCMV5p/EcoRI:5′-CGGAATTCCGCCGCCAGGGATG-3′ and NefCMV3p/HindIII: 5′-GCAAGCTTGCAGTT-3′. Nef^wt^ and Nef mutant vectors used for 293T transfections, including the negative control, were generated by O. S using pCMV-Nef [30].

### Transient transfections

Transfections were performed using calcium phosphate method [31]. Briefly, 10 million cells were plated in 100 mm^2^ plates in 10 ml of complete DMEM (10% FCS) 24 hours before transfection. At the day of transfection, medium was replaced by 10 ml of fresh warm complete DMEM (10% FCS). For each condition, 15–45 µg of DNA was mixed with 500 µl of CaCl_2_ (0.025 M). This step was followed by the addition of a 1∶1 mixture of BBS (BES buffered solution) previously incubated at room temperature for 20 minutes and then added to cells. BBS 2X was prepared by adding 50 mM N,Nbis (2-hydroxyethyl)-2-aminoethanesulfonic acid (BES; Calbiochem), 280 mM NaCl, 1.5 mM Na_2_HPO_4_ pH 6.95. Cells were harvested at 48 hours after transfection for either flow cytometry or Western blotting. We have routinely monitored our transfection efficacy by including irrelevant GFP plasmid; the transfection efficiency was always stable between 80–90%. The transfection efficiency of single transfected proteins was routinely monitored by determining the protein expression levels by Western blot analysis and normalization to the housekeeping gene *β*-actin.

### Biochemical analyses of Nef, CD4 and CTLA-4 proteins

Cells were lysed in lysis buffer containing 250 mM NaCl, 0.5% NP-40, 50 mM Hepes, 5 mM EDTA and protease inhibitors (Roche). Purified proteins were then quantified using the BCA kit (PIERCE). Cell lysates were then ran on 12% SDS gel and transferred on polyvinylidene fluoride (PVDF) membrane for Western blot analysis. For anti-CTLA-4 and anti-Nef blots, the primary antibodies used were homemade rabbit polyclonal antibodies followed by a step that includes incubation with a secondary goat anti-rabbit antibody conjugated to horseradish peroxidase (HRP). Bands were visualized by ECL (Amersham) then quantified by densitometric analysis using GelEval 1.22 software on images generated from films. The relative intensity of each band was normalized against *β*-actin or GAPDH.

### Confocal Microscopy

Cells were plated at 50,000 cells per coverslip mounted with poly-L-Lysin (Sigma) in 6-well plates. Transient transfections were carried out on these cells on coverslips using pCDNA3/CTLA-4 and/or pCMV/Nef-FLAG vectors. Forty-eight hours after transfection, cells on coverslips were washed with PBS containing 1 mM MgCl_2_ and fixed with 4% paraformaldehyde (PFA) for 15 min at room temperature. Cells were then washed by PBS and permeabilized by PBS containing 0.2% triton X-100 for 10 min at room temperature. After washings, cells were incubated with the primary and secondary antibodies as indicated in the figure legends. For CTLA-4 staining, 5 µl of anti-CTLA-4 and 1/50 dilution of secondary antibodies were used. For anti-FLAG, 1 µg of primary antibody and 1/50 secondary were used. Slides were then mounted and the images were taken with an LSM 510 META laser scanning confocal microscope (Zeiss) and 63 Plan-Apochromat objective with a numerical aperture of 1.4. Images were analyzed using Image J software (National Institute of Health).

### Flow cytometry

To monitor CTLA-4 expression on 293T cells transfected with CTLA-4, cells were stained with biotinylated goat anti-CTLA-4 antibody for 30 min at 4°C then washed twice with PBS-FBS (2%) and stained with Streptavidine-APC for 20 min (4°C). For CD4 expression, cells were stained with anti-CD4 PE antibody for 30 min at 4°C. Stained cells were then washed twice with PBS-FBS (2%) and fixed with 2% paraformaldehyde (in PBS) and analyzed on BD LSRII flow cytometer.

### Statistical Analysis

All *p* values were calculated by paired *t* test (two tailed). Pearson's correlation coefficient (R*r*) was used to determine the co-localization, segregation or lack of correlation for CTLA-4 and Nef. Pearson's correlation coefficient was obtained for each individual cell. In Pearson's correlation, the average pixel intensity values are subtracted from the original intensity values. As a result, the value of this coefficient ranges from −1 to 1, with a value of −1 representing a total lack of overlap between pixels from the images; a value of 1 indicates perfect image registration [32]. A single sample *t* test using GraphPad Prism software was performed to establish whether the correlation coefficient mean value was significantly different from zero [33].

## Results

### HIV-1 Nef protein down-regulates CTLA-4

**Figure 1:**
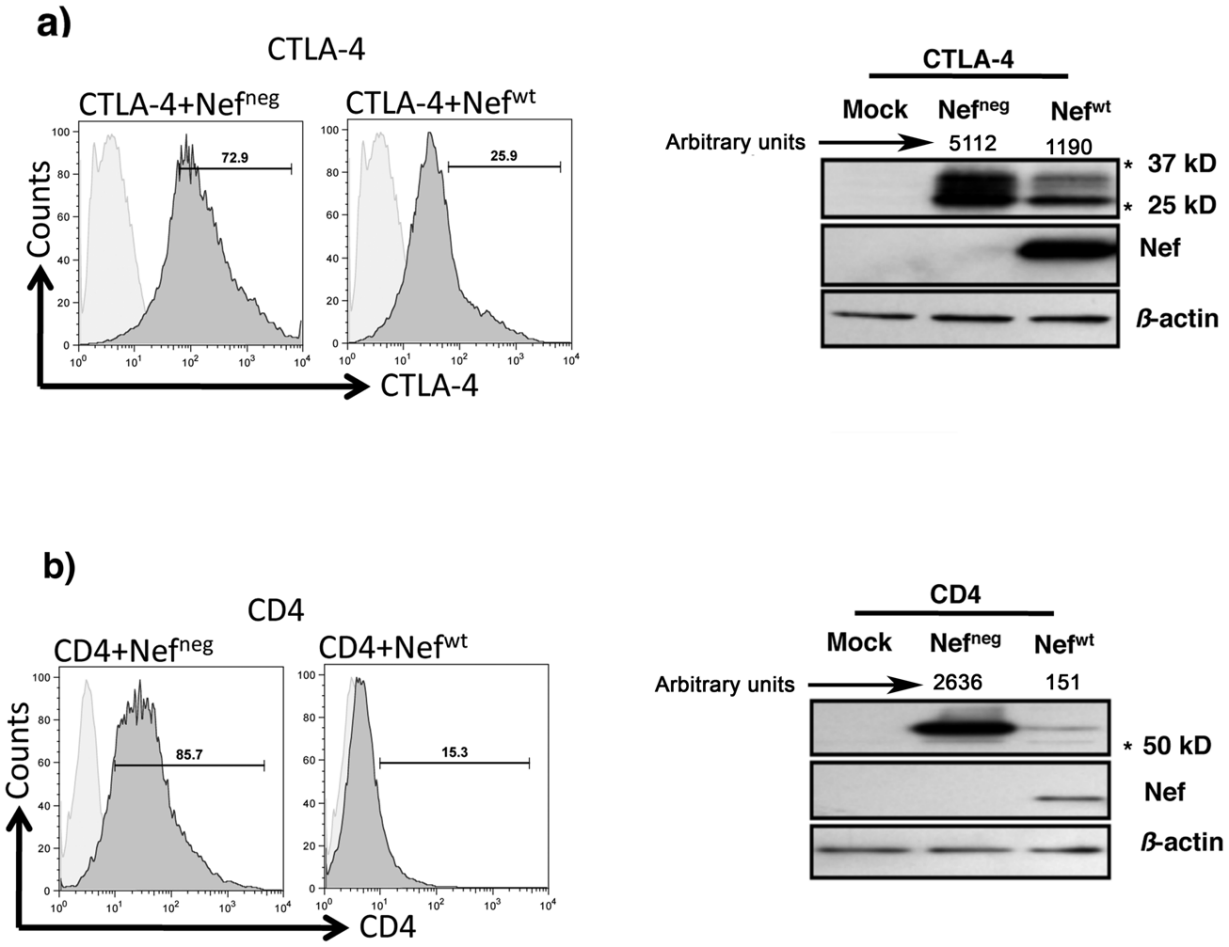
Nef-mediated CTLA-4 down-regulation. Transient co-expression of CTLA-4 and Nef in 293T cells. a) Left panels: Surface expression of CTLA-4 analyzed by FACS following staining of 293T cells co-transfected with CTLA-4 and either Nef^neg^ or Nef^wt^ vectors. Right panel: Western blotting analysis on total cell lysates from 293T cells mock-transfected or co-transfected with CTLA-4 and either Nef^neg^ or Nef^wt^ vectors (Arbitrary Densitometric units normalized to *ß-*actin are shown above corresponding lanes. b) Co-transfection of 293T cells with CD4 and either Nef^neg^ or Nef^wt^ vectors (Left and Right Panels as shown in a). * Refers to the corresponding MW on the SDS gel.

To assess the role of HIV-1 Nef protein in down-regulating CTLA-4 expression we established a transient transfection system to co-express human CTLA-4 and HIV-1 Nef (Nef^wt^) in 293T cells. A plasmid encompassing Nef in its reverse orientation (Nef^neg^) was used as negative control. Expression of Nef protein in CTLA-4-expressing 293T cells reduced CTLA-4 surface levels by 57–77% (n = 5) compared to cells transfected with Nef^neg^ plasmid (**Figure 1a** **, left panels**). Similarly, co-expression of Nef and CD4 in 293T cells led, as expected, to significant down-regulation of CD4 surface levels (80%) (**Figure 1b** **, left panels**). Nef-mediated down-regulation of both CTLA-4 and CD4 was also established by co-transfecting 293T cells with a GFP-reporter Nef construct followed by gating on GFP^+^ cells and determining the expression levels of CTLA-4 or CD4 (data not shown). Western blot analysis of total cell lysates was then used to assess whether CTLA-4 and CD4 down-regulation by Nef was mediated by protein degradation *versus* accelerated internalization and/or intracellular retention. As shown in **Figure 1a&b** **(right panels)** expression of Nef significantly decreased the total pools of both CTLA-4 and CD4 proteins, most likely by mediating protein degradation (the two forms of CTLA-4 on the CTLA-4 blot, **Figure 1a** **right panel**, correspond to membrane (30 kD) and cytosolic moieties (34 kD) [34]).

**Figure 2:**
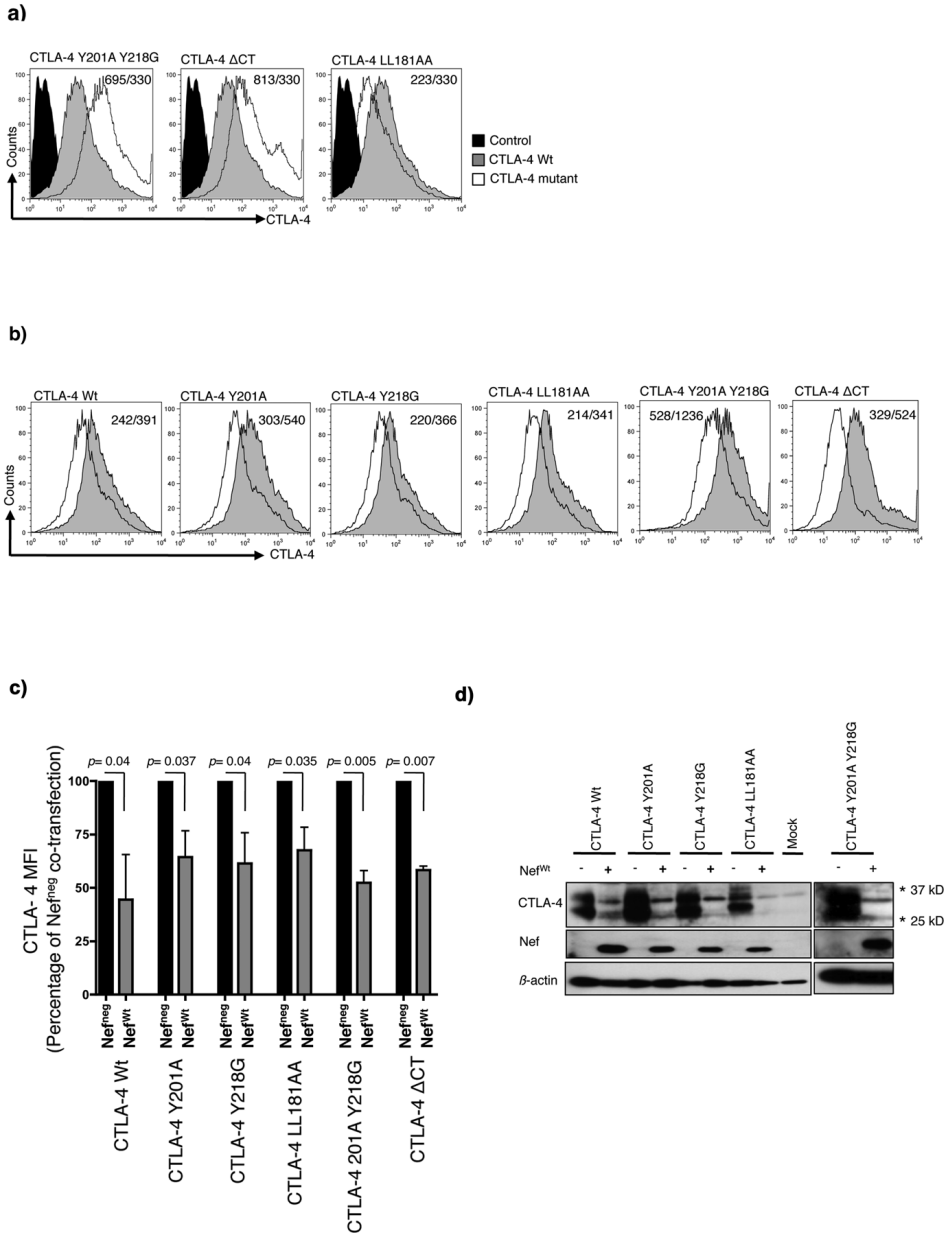
The cytoplasmic tail of CTLA-4 is dispensable for Nef-induced CTLA-4 down-regulation. a) Accumulation of CTLA-4 on the cell surface after transfection with CTLA-4 Y201AY218G and CTLA-4ΔCT mutants and lower expression after transfection with CTLA-4 LL181AA. Black solid histograms represent the isotype controls. Grey solid histograms represent the expression of CTLA-4 Wt. Black empty histograms represent CTLA-4 mutants. Numbers in inset represent the MFI of CTLA-4 mutant/CTLA-4 Wt. b) Surface expression levels of the different mutants of CTLA-4 with (empty histograms) or without (filled histograms) Nef expression as measured by flow cytometry. The numbers in inset represent the MFI of CTLA-4 under Nef^wt^/Nef^neg^ co-expression. c) MFI of surface CTLA-4 analyzed by FACS under Nef^neg^ or Nef^wt^ co-expression (mean of three independent experiments). The *p* values were calculated comparing values of CTLA-4 MFI under Nef^wt^ co-transfection relative to Nef^neg^ co-transfection. d) Western blot analysis showing the total expressions of CTLA-4 Wt and CTLA-4 mutated forms after co-transfection with Nef^neg^ (−) or Nef^wt^ (+) in 293T cells. * Refers to the corresponding MW on the SDS gel.

To test the hypothesis that Nef interacted with the cytoplasmic tail of CTLA-4 leading to Nef-mediated down-regulation of CTLA-4, we generated several CTLA-4 mutants known to impact on CTLA-4 cellular localization, trafficking and degradation. CTLA-4 Y201A and CTLA-4 Y218G mutants are unable to bind to the adaptor protein-2 (AP-2) and are expressed primarily on the cell surface and to a much lesser extent in intracellular compartments [35], [36]. These tyrosine motifs of CTLA-4 cytoplasmic tail were targeted by site directed mutagenesis to generate Y201A, Y218G and a double tyrosine mutated form Y201A Y218G. Another potential sorting double-leucine motif of CTLA-4 cytoplasmic tail LL181 was mutated to generate the LL181AA. We also generated a construct encompassing the CTLA-4 molecule deleted of its cytoplasmic tail (CTLA-4ΔCT). Mutating the tyrosine motifs or deleting the cytoplasmic tail resulted in the expected accumulation of CTLA-4 on the cell surface, due to lack of internalization signals [26], whereas the LL181AA mutant decreased CTLA-4 surface expression (**Figure 2a**). To determine the ability of Nef to down-regulate these mutated forms of CTLA-4 we co-transfected 293T cells with Nef^wt^ and CTLA-4 mutants. Our data shown in **Figure 2** (**b–d**) demonstrated that neither the tyrosine sorting motifs (CTLA-4 Y201A, CTLA-4 Y218G or CTLA-4 Y201AY218G) or the mutated leucine (CTLA-4 LL181AA) nor the deleted cytoplasmic tail construct (CTLA-4ΔCT) were able to rescue CTLA-4 expression upon co-transfection with Nef^wt^, as shown by FACS (**Figure 2b&c**) or by Western blotting (**Figure 2d**). There were no significant differences in the levels of CTLA-4 down-regulation between the CTLA-4 wt molecule and the different CTLA-4 mutants. Of note, the CTLA-4ΔCT mutant lost reactivity to the polyclonal antibody used for the Western blotting while retaining reactivity for antibodies used for the FACS analysis. These experiments show that increasing the levels of CTLA-4 by deleting or mutating the cytoplasmic tail did not affect the capacity of Nef to down-regulate this molecule. Thereby demonstrating that Nef does not require the CTLA-4 cytoplasmic tail to exert its effect. Our data with the CTLA-4ΔCT suggests that Nef down-regulates CTLA-4 expression mostly by interfering with CTLA-4 localization in intracellular compartments before reaching the plasma membrane instead of causing CTLA-4 internalization.

### Nef-mediated CTLA-4 down-regulation requires motifs in Nef involved in the interaction with the vacuolar ATPase, β-COP and the AP-1 sorting complexes

**Figure 3:**
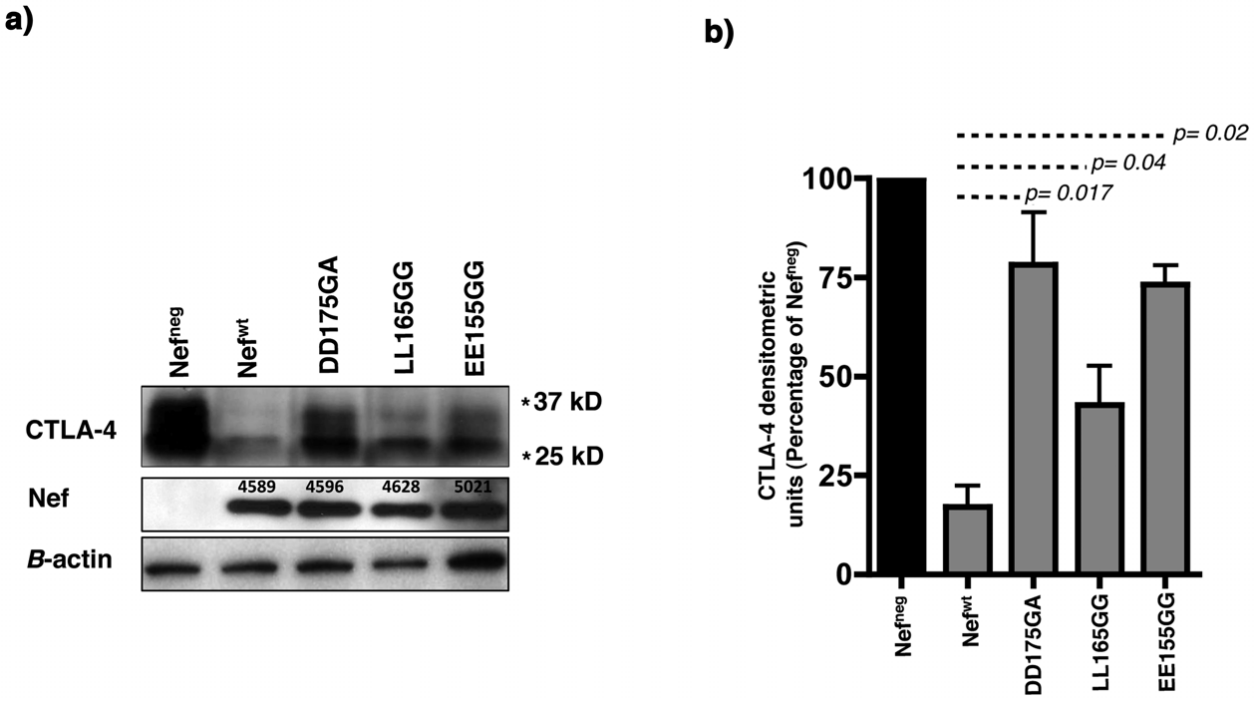
CTLA-4 down-regulation by Nef requires motifs in Nef involved in the interaction with the vacuolar ATPase, *β*-COP and the AP-1 sorting complexes. a) Total expression levels of CTLA-4 in 293T cells after co-transfection with Nef^wt^ or Nef mutants by Western blot. Numbers above the Nef blot represent the Nef densitometric units normalized to the *ß-*actin and showing similar expression levels for the different Nef constructs. b) Densitometric results, normalized to the *ß-*actin, are presented as mean percentages of CTLA-4 expression after co-transfection with Nef^wt^ or Nef mutants and normalization to the negative control (Nef^neg^) from 3 independent experiments. The *p* values are calculated using the percentage of CTLA-4 expression under co-transfection with each of the Nef mutants relative to CTLA-4 expression under Nef^wt^ co-transfection. * Refers to the corresponding MW on the SDS gel.

CTLA-4 traffics rapidly between intracellular vesicles and the plasma membrane, which accounts for its transient low levels of cell surface expression [37]. We tested a set of Nef mutants known to interfere with this protein trafficking in intracellular compartments [38]. The DD175 motif is required for the interaction of Nef with the v-ATPase, an enzyme present in intracellular vesicles that plays an important role in the maintenance of acidic pH of vesicular compartments, an essential component of the intracellular proteolytic machinery [39]. The EE155 motif is required for the interaction of Nef with the Coatomer subunit *ß*-COP, known to be involved in the transport of proteins from the ER to the *cis*-Golgi compartment [40]. This motif impacts on the late stages of Nef-dependent CD4 down-regulation [7]. The dileucine motif LL165 mediates the interaction of Nef with the sorting adaptor protein AP-1 [6]. To investigate the involvement of these pathways in the down-modulation of CTLA-4 by Nef, we co-transfected CTLA-4 and the above listed Nef mutant constructs into 293T cells. Our Western blot analysis on total lysates from these cells (**Figure 3a**) showed that these Nef mutants, DD175GA, LL165GG and EE155GG had a significantly lower ability to mediate CTLA-4 degradation compared to Nef^wt^ (*p* = 0.017, 0.04 and 0.02, respectively, **Figure 3b** **, n = 3**). However, among the three Nef mutants, LL165GG had a higher capacity to down-regulate CTLA-4 compared to DD175GA and EE155GG (57% *vs* 22 and 26% mediated CTLA-4 down-regulation, respectively).

Altogether, these data indicated that Nef down-modulates CTLA-4 through a mechanism that requires the interaction of Nef with the sorting adaptor protein AP-1, the v-ATPase as well as with the *β*-COP subunit. This also suggested that cellular compartments where interaction between CTLA-4 and Nef takes place are likely to be endosomes and lysosomes, where the v-ATPase is active [41].

### Lysosomal function is required for Nef-induced down-regulation of CTLA-4

**Figure 4:**
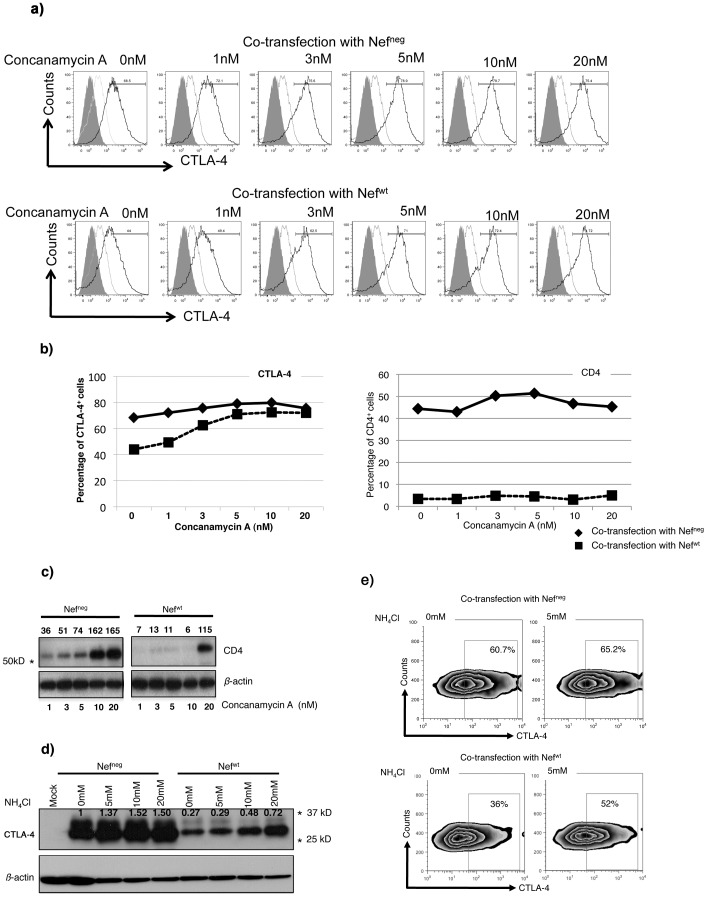
Lysosomal function is required for Nef-induced down-regulation of CTLA-4. a) CTLA-4 surface expression levels on 293T cells co-transfected with Nef^neg^ (upper panels) or Nef^wt^ (lower panels) after treatment with increasing concentrations of Concanamycin A. Grey solid histograms represent the unstained controls. Grey empty histograms represent Mock-transfected cells (stained with anti-CTLA-4 antibody) and Solid Black histograms represent CTLA-4 and either Nef^neg^ (upper panels) or Nef^wt^ (lower panels) co-transfected cells. b) Percentage of CTLA-4^+^ cells (left panel) and CD4^+^ cells (right panel) from the same experiment at different concentrations of Concanamycin A (0–20 nM). Solid line represents CTLA-4 or CD4 under Nef^neg^ and dotted line represents CTLA-4 or CD4 under Nef^wt^ co-transfection. c) Rescue of total CD4 protein levels by Concanamycin A treatment. Total lysates from 293T cells co-transfected with CD4 and Nef^neg^ or Nef^wt^ and treated with increasing concentrations of Concanamycin A (1–20 nM). Numbers on top of CD4 blot represent arbitrary densitometric units after normalization to the *β*-actin. d) Western blot analysis for total lysates from 293T cells co-transfected with CTLA-4 and either Nef^neg^ or Nef^wt^ vectors and treated with increasing concentrations of NH_4_Cl (0–20 mM) (n = 2). Numbers on the CTLA-4 Western blot represent arbitrary units for CTLA-4 protein expression levels after normalization to *β*-actin. e) FACS analysis on 293T cells showing CTLA-4 surface levels under Nef^neg^ (upper panels) and Nef^wt^ (lower panels) co-expression in the presence or absence of NH_4_Cl. * Refers to the corresponding MW on the SDS gel.

Results in Figure 3 suggested that Nef-induced CTLA-4 down-regulation was likely mediated by protein degradation in lysosomes since mutation of the v-ATPase-binding motif of Nef (DD175GA) rescued CTLA-4 expression. To confirm the role of acidic compartments in Nef-induced CTLA-4 down-regulation, we investigated the impact of Nef on CTLA-4 expression in the presence of Concanamycin A, a specific inhibitor of the vacuolar ATPase [42]. Similar experiments were performed in the presence of the weak-base amine ammonium chloride (NH_4_Cl) known to inhibit the lysosomal machinery by increasing the pH of endocytic and lysosomal vesicles [43], [44], [45]. 293T cells co-transfected with CTLA-4 (or CD4) and Nef^wt^ were treated with increasing concentrations of Concanamycin A or NH_4_Cl. Treatment of 293T cells expressing Nef^wt^ and CTLA-4 with 1, 3, 5, 10 and 20 nM Concanamycin A increased, in a dose-dependent manner, the levels of CTLA-4 expression as measured by the frequency of CTLA-4^+^ cells, from 44% to 72% (**Figure 4a** **, lower panels &** **4b** **, left panel**). In contrast, treatment with Concanamycin A of 293T cells co-transfected with CD4 and Nef^wt^ did not rescue CD4 surface expression (**Figure 4b** **, right panel**). However, at higher concentrations (20 nM) of Concanamycin A, increased-expression of CD4 protein was only observed with biochemical analysis on total cell lysates (**Figure 4c**) suggesting the accumulation of CD4 in intracellular compartments, consistent with earlier observations by other groups [46].

Ammonium chloride, a lysosomal inhibitor, treatment of 293T cells expressing Nef^wt^ and CTLA-4 also increased the total pool of CTLA-4 by 2.7 fold (**Figure 4d** **for Western blotting and 4e for surface expression by FACS analysis**). Of note, we also observed a dose-dependent accumulation of the total molecular pool of CTLA-4 in cells co-transfected with Nef^neg^ vector treated with either Concanamycin A (**Figure 4a** **, upper panels**) or with NH_4_Cl (**Figure 4d&e**), most likely resulting from the inhibition of steady-state degradation of CTLA-4. As expected, the expression of both CTLA-4 and CD4 was indeed down-regulated in this transfection system in the absence of both inhibitors (**Figure 4a–e**).

Altogether, our results showed that CTLA-4 is down-regulated by lysosomal degradation as previously shown for CD4. However, we provide evidence herein that significant differences exist in the mechanisms leading to the down-regulation of the two molecules as CTLA-4 re-circulates to the cell surface following treatment with lysosomal inhibitors whereas, CD4 does not and likely accumulates in intracellular compartments.

### Nef and CTLA-4 co-localize in early and recycling endosomes

**Figure 5:**
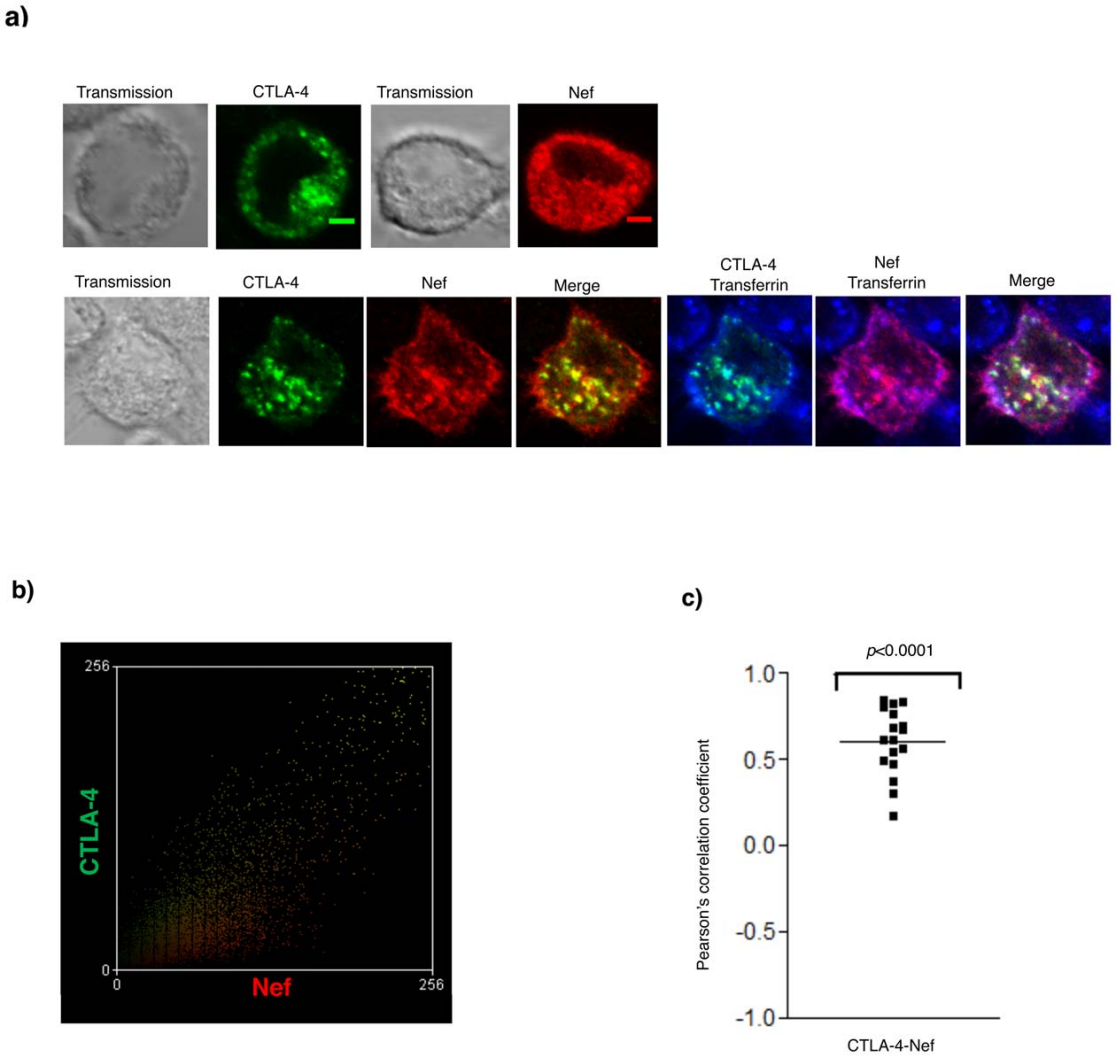
Nef and CTLA-4 co-localize in early and recycling endosomes. a) HeLa cells transfected only with a FLAG-Nef expression vector (upper panels) or co-transfected (lower panels) with CTLA-4 expressing vector were incubated with Alexa 633 labeled transferrin (blue), CTLA-4 specific antibody (green) and FLAG-Nef antibody (red). The extreme left panels show a transmission light field. The middle lower panel shows the co-localization between Nef and CTLA-4 (yellow color results from merging green (CTLA-4) and red (Nef)). The extreme right lower panel shows the co-localization of the three proteins; CTLA-4, Nef and transferrin together (the white color is a combination of green (CTLA-4), red (Nef) and blue (transferrin)), Bar = 20 µm. b) Scatter diagram showing the intensity of the CTLA-4-Alexa-fluor 488 (Y-axis) and Nef-Alexa-fluor 546 (X-axis). c) Levels of co-localization between CTLA-4 and Nef is represented by the mean *Pearson's* correlation coefficient (R*r*) (n = 16, 9 fields).

The results described above suggested that Nef and CTLA-4 most likely co-localize in intracellular compartments where protein degradation typically occurs including lysosomes. In order to confirm this hypothesis, we monitored the intracellular localization of both proteins by confocal microscopy in transiently transfected HeLa cells. The transferrin receptor (Trf) was used as a marker for early and recycling endosomes [47], which is the compartment most likely involved in the initial Nef-CTLA-4 interaction. CTLA-4 is known to reside in intracellular endocytic compartments prior to its translocation to the plasma membrane [48]. Confocal microscopy analysis confirmed the presence of CTLA-4 in specific intracellular granules (endosomes) where the Trf, a marker of early endosomes [47], is expressed (**Figure 5a**). As expected from our mutagenesis studies, these Trf^+^ early and recycling endosomes showed significant co-localization between Nef and CTLA-4. The scatter diagram shows the intensity of fluorescence for CTLA-4 (Y axis) and Nef (X axis) in one single representative cell (**Figure 5b**). The white granules (**Figure 5a** **lower panels last picture**) depict the co-localization of Nef, CTLA-4 and Trf. Quantification of CTLA-4 and Nef co-localization was assessed in multiple independent cells (**Figure 5c**) (n = 16 and a *Pearson* coefficient value of 0.6, *p*<0.0001).

Previous results (**Figure 2**) showing that deletion of the cytoplasmic tail of CTLA-4 did not affect the capacity of Nef to down-regulate CTLA-4, indicated that the interaction between the two molecules occurs mostly in intracellular compartments. This was confirmed by confocal microscopy visualization, since the co-localization between CTLA-4 and Nef occurred mostly in intracellular compartments and to a lesser extent at the plasma membrane (yellow granules that indicate Nef (red) and CTLA-4 (green) interaction are primarily present in intracellular compartments). This was also different from what was described for CD4 as Nef was previously shown to interfere with CD4 expression at the plasma membrane [49]. Together, these results showed that Nef co-localizes with CTLA-4 in intracellular acidic compartments, most likely lysosomes, where accelerated degradation of CTLA-4 occurs.

## Discussion

In this study we provide first line of evidence that HIV Nef down-regulates CTLA-4 expression at the cell surface by interacting with CTLA-4 in intracellular compartments including endosomes and lysosomes. The down-regulation of CTLA-4 by Nef, early in acute infection when most activated T cells express CTLA-4 at the cell surface [50], [51] could provide an important mechanism to circumvent the physiological effect of CTLA-4 i.e. inhibition of T cell activation and HIV replication, and to allow viral dissemination.

Nef proteins from virtually all primate lentiviruses modulate the expression of a large number of cell surface proteins including CD4 [2], [52], [53], [54], [55], [56] and MHC I molecules [10], [57] through multiple mechanisms. We show here that Nef mediates CTLA-4 and CD4 down-regulation *via* distinct mechanisms. The CTLA-4 cytoplasmic tail was dispensable for this new function of Nef. In contrast, the CD4 cytoplasmic tail was shown to be required for CD4 degradation by Nef as CD4ΔCT or a chimera between the CD4 extracellular and the CD8 intracellular domains are resistant to Nef-induced down-regulation [53], [58]. We also showed that Nef interacts with CTLA-4 in intracellular compartments. This was confirmed by our confocal microscopy visualization showing that most Nef/CTLA-4 interactions occurred in early and recycling endosomes with very low levels of co-localization of these two molecules at the plasma membrane (Figure 5). The low levels of CTLA-4 at the cell surface could likely explain the lack of co-localization between CTLA-4 and Nef at the plasma membrane, although CTLA-4 mutants, known to be highly expressed at the cell surface, were still down-regulated by Nef to levels similar to those of the wt molecule. Our results suggest that CTLA-4 is directed for degradation in the endosomal/lysosomal compartment prior to reaching the plasma membrane, thus highlighting a major difference in the mechanisms that lead to Nef-mediated degradation of CTLA-4 *versus* CD4.

Despite the above listed differences, similarities were also observed in the mechanisms of Nef-mediated down-regulation of CTLA-4 and CD-4. Both mechanisms required Nef motifs known to be involved in vesicle trafficking. The double aspartic acid DD175 of Nef is involved in the interaction of Nef with the catalytic subunit of vacuolar ATPase (vATPase), originally known as Nef binding protein 1 or NBP1, an essential component of lysosome degradation machinery [55]. Binding to the vATPase was shown to be required for the successful interaction of Nef with the endocytic machinery by connecting Nef to the μ2 chain of AP-2 adaptor protein [59]. Similar to CD4 down-regulation, the DD175GA mutant completely abolished the capacity of Nef to modulate CTLA-4 expression, suggesting that the major mechanism leading to CTLA-4 down-regulation by Nef is protein degradation and not just retention in intracellular compartments. This is supported by our results showing that Nef expression leads to a major reduction in the total intracellular pool of CTLA-4 molecules. Moreover CTLA-4 expression was indeed rescued when weak-base amines were used to neutralize the lysosomal acidity or to inhibit the vATPase activity (**Figure 4**). Importantly, in contrast to CD4, treatment with pH-neutralizing agents rescued CTLA-4 surface expression, thus highlighting another difference between Nef-mediated down-regulation of CD4 and CTLA-4.

Together, our study reveals a novel potential mechanism for HIV pathogenesis by which Nef-mediated CTLA-4 down-regulation may decrease the threshold of T cell activation, a critical step for HIV-1 replication and dissemination. Nef-mediated down-regulation of CTLA-4 on the cell surface of infected cells may also contribute to the global hyperimmune activation, a hallmark of HIV infection. In line with, Nef was shown to be exported from infected cells through accelerated release of Nef-containing exosomes [60]. Extracellular Nef targets bystander CD4^+^ T cells for apoptosis [61] and also B cells for the suppression of immunoglobulin class switching [62]. The uptake of Nef by bystander CD4^+^ T cells may result in the down-regulation of CTLA-4 on the cell surface from non-infected cells leading to global sustained hyperimmune activation and increased viral replication. Interestingly, work by Cecchinato et al. [63] on SIV-infected non-human primates (NPHs) demonstrated that antagonizing CTLA-4 effect by blocking CTLA-4 with specific antibodies prior to and following SIV challenge led to hyperimmune activation and increased viral replication. Most importantly, this treatment decreased the responsiveness to antiretroviral therapy and abrogated the ability of therapeutic T cell vaccines to decrease the viral load set point. This effect is likely a consequence of the lack of CTLA-4 binding to its ligands leading to substantial increase of activated CD4^+^ T cells and subsequent infection with SIV. Therefore, CTLA-4 evidently plays a physiological role in limiting viral replication and/or dissemination and that Nef-mediated down-regulation of CTLA-4 is likely to counteract this function.

In conclusion, our findings showing that HIV-1 Nef protein down-regulates the negative modulator CTLA-4 represent a novel mechanism for HIV-1 pathogenesis that is likely involved in the enhancement of T cell activation and T cell turnover, two key cellular functions that are important for HIV-1 replication and dissemination. HIV-1 Nef is subject to high sequence variation during the course of infection and among infected individuals. The ability of HV-1 Nef to modulate CTLA-4 expression may be different between viral strains and may be linked to the course of disease progression. Our study opens the path for this new type of investigation, with a particular focus on differences in HIV Nef immunoregulatory functions between transmitted/founder *versus* chronic viruses and between subjects with slow *versus* rapid disease progression.

## References

[1] Malim MH, Emerman M (2008) HIV-1 accessory proteins–ensuring viral survival in a hostile environment. Cell Host Microbe 3: 388–398. 18541215 10.1016/j.chom.2008.04.008

[2] Aiken C, Konner J, Landau NR, Lenburg ME, Trono D (1994) Nef induces CD4 endocytosis: requirement for a critical dileucine motif in the membrane-proximal CD4 cytoplasmic domain. Cell 76: 853–864. 8124721 10.1016/0092-8674(94)90360-3

[3] Arold ST, Baur AS (2001) Dynamic Nef and Nef dynamics: how structure could explain the complex activities of this small HIV protein. Trends Biochem Sci 26: 356–363. 11406408 10.1016/s0968-0004(01)01846-1

[4] Benichou S, Bomsel M, Bodeus M, Durand H, Doute M, et al (1994) Physical interaction of the HIV-1 Nef protein with beta-COP, a component of non-clathrin-coated vesicles essential for membrane traffic. J Biol Chem 269: 30073–30076. 7982906

[5] Chen BK, Gandhi RT, Baltimore D (1996) CD4 down-modulation during infection of human T cells with human immunodeficiency virus type 1 involves independent activities of vpu, env, and nef. J Virol 70: 6044–6053. 8709227 10.1128/jvi.70.9.6044-6053.1996 PMC190625

[6] Bresnahan PA, Yonemoto W, Ferrell S, Williams-Herman D, Geleziunas R, et al (1998) A dileucine motif in HIV-1 Nef acts as an internalization signal for CD4 downregulation and binds the AP-1 clathrin adaptor. Curr Biol 8: 1235–1238. 9811606 10.1016/s0960-9822(07)00517-9

[7] Faure J, Stalder R, Borel C, Sobo K, Piguet V, et al (2004) ARF1 regulates Nef-induced CD4 degradation. Curr Biol 14: 1056–1064. 15202998 10.1016/j.cub.2004.06.021

[8] Lundquist CA, Tobiume M, Zhou J, Unutmaz D, Aiken C (2002) Nef-mediated downregulation of CD4 enhances human immunodeficiency virus type 1 replication in primary T lymphocytes. J Virol 76: 4625–4633. 11932428 10.1128/JVI.76.9.4625-4633.2002 PMC155097

[9] Piguet V, Trono D (1999) The Nef protein of primate lentiviruses. Rev Med Virol 9: 111–120. 10386338 10.1002/(sici)1099-1654(199904/06)9:2<111::aid-rmv245>3.0.co;2-p

[10] Schwartz O, Marechal V, Le Gall S, Lemonnier F, Heard JM (1996) Endocytosis of major histocompatibility complex class I molecules is induced by the HIV-1 Nef protein. Nat Med 2: 338–342. 8612235 10.1038/nm0396-338

[11] Linnemann T, Zheng YH, Mandic R, Peterlin BM (2002) Interaction between Nef and phosphatidylinositol-3-kinase leads to activation of p21-activated kinase and increased production of HIV. Virology 294: 246–255. 12009866 10.1006/viro.2002.1365

[12] Arora VK, Molina RP, Foster JL, Blakemore JL, Chernoff J, et al (2000) Lentivirus Nef specifically activates Pak2. J Virol 74: 11081–11087. 11070003 10.1128/jvi.74.23.11081-11087.2000 PMC113188

[13] Wolf D, Witte V, Laffert B, Blume K, Stromer E, et al (2001) HIV-1 Nef associated PAK and PI3-kinases stimulate Akt-independent Bad-phosphorylation to induce anti-apoptotic signals. Nat Med 7: 1217–1224. 11689886 10.1038/nm1101-1217

[14] Lee CH, Saksela K, Mirza UA, Chait BT, Kuriyan J (1996) Crystal structure of the conserved core of HIV-1 Nef complexed with a Src family SH3 domain. Cell 85: 931–942. 8681387 10.1016/s0092-8674(00)81276-3

[15] Collette Y, Dutartre H, Benziane A, Ramos M, Benarous R, et al (1996) Physical and functional interaction of Nef with Lck. HIV-1 Nef-induced T-cell signaling defects. J Biol Chem 271: 6333–6341. 8626429 10.1074/jbc.271.11.6333

[16] Lee CH, Leung B, Lemmon MA, Zheng J, Cowburn D, et al (1995) A single amino acid in the SH3 domain of Hck determines its high affinity and specificity in binding to HIV-1 Nef protein. EMBO J 14: 5006–5015. 7588629 10.1002/j.1460-2075.1995.tb00183.x PMC394604

[17] Wu Y, Marsh JW (2001) Selective transcription and modulation of resting T cell activity by preintegrated HIV DNA. Science 293: 1503–1506. 11520990 10.1126/science.1061548

[18] Schrager JA, Marsh JW (1999) HIV-1 Nef increases T cell activation in a stimulus-dependent manner. Proc Natl Acad Sci U S A 96: 8167–8172. 10393966 10.1073/pnas.96.14.8167 PMC22206

[19] Kroczek RA, Mages HW, Hutloff A (2004) Emerging paradigms of T-cell co-stimulation. Curr Opin Immunol 16: 321–327. 15134781 10.1016/j.coi.2004.03.002

[20] Waterhouse P, Penninger JM, Timms E, Wakeham A, Shahinian A, et al (1995) Lymphoproliferative disorders with early lethality in mice deficient in Ctla-4. Science 270: 985–988. 7481803 10.1126/science.270.5238.985

[21] Carreno BM, Bennett F, Chau TA, Ling V, Luxenberg D, et al (2000) CTLA-4 (CD152) can inhibit T cell activation by two different mechanisms depending on its level of cell surface expression. J Immunol 165: 1352–1356. 10903737 10.4049/jimmunol.165.3.1352

[22] Wing K, Onishi Y, Prieto-Martin P, Yamaguchi T, Miyara M, et al (2008) CTLA-4 control over Foxp3+ regulatory T cell function. Science 322: 271–275. 18845758 10.1126/science.1160062

[23] Munn DH, Sharma MD, Mellor AL (2004) Ligation of B7-1/B7-2 by human CD4+ T cells triggers indoleamine 2,3-dioxygenase activity in dendritic cells. J Immunol 172: 4100–4110. 15034022 10.4049/jimmunol.172.7.4100

[24] Leung HT, Bradshaw J, Cleaveland JS, Linsley PS (1995) Cytotoxic T lymphocyte-associated molecule-4, a high-avidity receptor for CD80 and CD86, contains an intracellular localization motif in its cytoplasmic tail. J Biol Chem 270: 25107–25114. 7559643 10.1074/jbc.270.42.25107

[25] Linsley PS, Bradshaw J, Greene J, Peach R, Bennett KL, et al (1996) Intracellular trafficking of CTLA-4 and focal localization towards sites of TCR engagement. Immunity 4: 535–543. 8673700 10.1016/s1074-7613(00)80480-x

[26] Chuang E, Lee KM, Robbins MD, Duerr JM, Alegre ML, et al (1999) Regulation of cytotoxic T lymphocyte-associated molecule-4 by Src kinases. J Immunol 162: 1270–1277. 9973379

[27] Krummel MF, Allison JP (1996) CTLA-4 engagement inhibits IL-2 accumulation and cell cycle progression upon activation of resting T cells. J Exp Med 183: 2533–2540. 8676074 10.1084/jem.183.6.2533 PMC2192613

[28] Guntermann C, Alexander DR (2002) CTLA-4 suppresses proximal TCR signaling in resting human CD4(+) T cells by inhibiting ZAP-70 Tyr(319) phosphorylation: a potential role for tyrosine phosphatases. J Immunol 168: 4420–4429. 11970985 10.4049/jimmunol.168.9.4420

[29] Harlin H, Hwang KW, Palucki DA, Kim O, Thompson CB, et al (2002) CTLA-4 engagement regulates NF-kappaB activation in vivo. Eur J Immunol 32: 2095–2104. 12209621 10.1002/1521-4141(200208)32:8<2095::AID-IMMU2095>3.0.CO;2-E

[30] Bachelerie F, Alcami J, Hazan U, Israel N, Goud B, et al (1990) Constitutive expression of human immunodeficiency virus (HIV) nef protein in human astrocytes does not influence basal or induced HIV long terminal repeat activity. J Virol 64: 3059–3062. 2186177 10.1128/jvi.64.6.3059-3062.1990 PMC249492

[31] Jordan M, Schallhorn A, Wurm FM (1996) Transfecting mammalian cells: optimization of critical parameters affecting calcium-phosphate precipitate formation. Nucleic Acids Res 24: 596–601. 8604299 10.1093/nar/24.4.596 PMC145683

[32] Manders EM, Stap J, Brakenhoff GJ, van Driel R, Aten JA (1992) Dynamics of three-dimensional replication patterns during the S-phase, analysed by double labelling of DNA and confocal microscopy. J Cell Sci 103 Pt 3: 857–862. 1478975 10.1242/jcs.103.3.857

[33] Tseng SY, Waite JC, Liu M, Vardhana S, Dustin ML (2008) T cell-dendritic cell immunological synapses contain TCR-dependent CD28-CD80 clusters that recruit protein kinase C theta. J Immunol 181: 4852–4863. 18802089 10.4049/jimmunol.181.7.4852 PMC2556893

[34] Chun T, Choi HJ, Chung YH (2004) Two different forms of human CTLA-4 proteins following peripheral T cell activation. Immunol Lett 91: 213–220. 15019292 10.1016/j.imlet.2003.12.004

[35] Shiratori T, Miyatake S, Ohno H, Nakaseko C, Isono K, et al (1997) Tyrosine phosphorylation controls internalization of CTLA-4 by regulating its interaction with clathrin-associated adaptor complex AP-2. Immunity 6: 583–589. 9175836 10.1016/s1074-7613(00)80346-5

[36] Zhang Y, Allison JP (1997) Interaction of CTLA-4 with AP50, a clathrin-coated pit adaptor protein. Proc Natl Acad Sci U S A 94: 9273–9278. 9256472 10.1073/pnas.94.17.9273 PMC23153

[37] Mead KI, Zheng Y, Manzotti CN, Perry LC, Liu MK, et al (2005) Exocytosis of CTLA-4 is dependent on phospholipase D and ADP ribosylation factor-1 and stimulated during activation of regulatory T cells. J Immunol 174: 4803–4811. 15814706 10.4049/jimmunol.174.8.4803

[38] Fackler OT, Moris A, Tibroni N, Giese SI, Glass B, et al (2006) Functional characterization of HIV-1 Nef mutants in the context of viral infection. Virology 351: 322–339. 16684552 10.1016/j.virol.2006.03.044

[39] Stevens TH, Forgac M (1997) Structure, function and regulation of the vacuolar (H+)-ATPase. Annu Rev Cell Dev Biol 13: 779–808. 9442887 10.1146/annurev.cellbio.13.1.779

[40] Peter F, Plutner H, Zhu H, Kreis TE, Balch WE (1993) Beta-COP is essential for transport of protein from the endoplasmic reticulum to the Golgi in vitro. J Cell Biol 122: 1155–1167. 8376457 10.1083/jcb.122.6.1155 PMC2119854

[41] Mindell JA (2012) Lysosomal acidification mechanisms. Annu Rev Physiol 74: 69–86. 22335796 10.1146/annurev-physiol-012110-142317

[42] Huss M, Ingenhorst G, Konig S, Gassel M, Drose S, et al (2002) Concanamycin A, the specific inhibitor of V-ATPases, binds to the V(o) subunit c. J Biol Chem 277: 40544–40548. 12186879 10.1074/jbc.M207345200

[43] Maxfield FR (1982) Weak bases and ionophores rapidly and reversibly raise the pH of endocytic vesicles in cultured mouse fibroblasts. J Cell Biol 95: 676–681. 6183281 10.1083/jcb.95.2.676 PMC2112942

[44] Ohkuma S, Poole B (1978) Fluorescence probe measurement of the intralysosomal pH in living cells and the perturbation of pH by various agents. Proc Natl Acad Sci U S A 75: 3327–3331. 28524 10.1073/pnas.75.7.3327 PMC392768

[45] Jeong JY, Choi JW, Jeon KI, Jue DM (2002) Chloroquine decreases cell-surface expression of tumour necrosis factor receptors in human histiocytic U-937 cells. Immunology 105: 83–91. 11849318 10.1046/j.0019-2805.2001.01339.x PMC1782639

[46] Luo T, Anderson SJ, Garcia JV (1996) Inhibition of Nef- and phorbol ester-induced CD4 degradation by macrolide antibiotics. J Virol 70: 1527–1534. 8627671 10.1128/jvi.70.3.1527-1534.1996 PMC189974

[47] Duprez V, Smoljanovic M, Lieb M, Dautry-Varsat A (1994) Trafficking of interleukin 2 and transferrin in endosomal fractions of T lymphocytes. J Cell Sci 107 Pt 5: 1289–1295. 7929635 10.1242/jcs.107.5.1289

[48] Alegre ML, Noel PJ, Eisfelder BJ, Chuang E, Clark MR, et al (1996) Regulation of surface and intracellular expression of CTLA4 on mouse T cells. J Immunol 157: 4762–4770. 8943377

[49] Cluet D, Bertsch C, Beyer C, Gloeckler L, Erhardt M, et al (2005) Detection of human immunodeficiency virus type 1 Nef and CD4 physical interaction in living human cells by using bioluminescence resonance energy transfer. J Virol 79: 8629–8636. 15956605 10.1128/JVI.79.13.8629-8636.2005 PMC1143710

[50] Kaufmann DE, Kavanagh DG, Pereyra F, Zaunders JJ, Mackey EW, et al (2007) Upregulation of CTLA-4 by HIV-specific CD4+ T cells correlates with disease progression and defines a reversible immune dysfunction. Nat Immunol 8: 1246–1254. 17906628 10.1038/ni1515

[51] Leng Q, Bentwich Z, Magen E, Kalinkovich A, Borkow G (2002) CTLA-4 upregulation during HIV infection: association with anergy and possible target for therapeutic intervention. AIDS 16: 519–529. 11872994 10.1097/00002030-200203080-00002

[52] Garcia JV, Miller AD (1991) Serine phosphorylation-independent downregulation of cell-surface CD4 by nef. Nature 350: 508–511. 2014052 10.1038/350508a0

[53] Harris MP, Neil JC (1994) Myristoylation-dependent binding of HIV-1 Nef to CD4. J Mol Biol 241: 136–142. 8057354 10.1006/jmbi.1994.1483

[54] Hua J, Blair W, Truant R, Cullen BR (1997) Identification of regions in HIV-1 Nef required for efficient downregulation of cell surface CD4. Virology 231: 231–238. 9168885 10.1006/viro.1997.8517

[55] Lu X, Yu H, Liu SH, Brodsky FM, Peterlin BM (1998) Interactions between HIV1 Nef and vacuolar ATPase facilitate the internalization of CD4. Immunity 8: 647–656. 9620685 10.1016/s1074-7613(00)80569-5

[56] Liu LX, Heveker N, Fackler OT, Arold S, Le Gall S, et al (2000) Mutation of a conserved residue (D123) required for oligomerization of human immunodeficiency virus type 1 Nef protein abolishes interaction with human thioesterase and results in impairment of Nef biological functions. J Virol 74: 5310–5319. 10799608 10.1128/jvi.74.11.5310-5319.2000 PMC110886

[57] Piguet V, Gu F, Foti M, Demaurex N, Gruenberg J, et al (1999) Nef-induced CD4 degradation: a diacidic-based motif in Nef functions as a lysosomal targeting signal through the binding of beta-COP in endosomes. Cell 97: 63–73. 10199403 10.1016/s0092-8674(00)80715-1

[58] Garcia JV, Alfano J, Miller AD (1993) The negative effect of human immunodeficiency virus type 1 Nef on cell surface CD4 expression is not species specific and requires the cytoplasmic domain of CD4. J Virol 67: 1511–1516. 8437228 10.1128/jvi.67.3.1511-1516.1993 PMC237521

[59] Geyer M, Yu H, Mandic R, Linnemann T, Zheng YH, et al (2002) Subunit H of the V-ATPase binds to the medium chain of adaptor protein complex 2 and connects Nef to the endocytic machinery. J Biol Chem 277: 28521–28529. 12032142 10.1074/jbc.M200522200

[60] Lenassi M, Cagney G, Liao M, Vaupotic T, Bartholomeeusen K, et al (2010) HIV Nef is secreted in exosomes and triggers apoptosis in bystander CD4+ T cells. Traffic 11: 110–122. 19912576 10.1111/j.1600-0854.2009.01006.x PMC2796297

[61] James CO, Huang MB, Khan M, Garcia-Barrio M, Powell MD, et al (2004) Extracellular Nef protein targets CD4+ T cells for apoptosis by interacting with CXCR4 surface receptors. J Virol 78: 3099–3109. 14990729 10.1128/JVI.78.6.3099-3109.2004 PMC353732

[62] Qiao X, He B, Chiu A, Knowles DM, Chadburn A, et al (2006) Human immunodeficiency virus 1 Nef suppresses CD40-dependent immunoglobulin class switching in bystander B cells. Nat Immunol 7: 302–310. 16429138 10.1038/ni1302

[63] Cecchinato V, Tryniszewska E, Ma ZM, Vaccari M, Boasso A, et al (2008) Immune activation driven by CTLA-4 blockade augments viral replication at mucosal sites in simian immunodeficiency virus infection. J Immunol 180: 5439–5447. 18390726 10.4049/jimmunol.180.8.5439 PMC2768121

